# Future changes in extreme weather and pyroconvection risk factors for Australian wildfires

**DOI:** 10.1038/s41598-019-46362-x

**Published:** 2019-07-11

**Authors:** Andrew J. Dowdy, Hua Ye, Acacia Pepler, Marcus Thatcher, Stacey L. Osbrough, Jason P. Evans, Giovanni Di Virgilio, Nicholas McCarthy

**Affiliations:** 1 Climate Research Section, Bureau of Meteorology, Melbourne, Australia; 2 CSIRO, Melbourne, Australia; 3 Climate Change Research Centre, University of New South Wales, Sydney, Australia; 4 Australian Research Council Centre of Excellence for Climate Extremes, University of New South Wales, Sydney, Australia; 5 University of Queensland, Brisbane, Australia

**Keywords:** Atmospheric dynamics, Climate and Earth system modelling, Projection and prediction, Natural hazards

## Abstract

Extreme wildfires have recently caused disastrous impacts in Australia and other regions of the world, including events with strong convective processes in their plumes (i.e., strong pyroconvection). Dangerous wildfire events such as these could potentially be influenced by anthropogenic climate change, however, there are large knowledge gaps on how these events might change in the future. The McArthur Forest Fire Danger Index (FFDI) is used to represent near-surface weather conditions and the Continuous Haines index (CH) is used here to represent lower to mid-tropospheric vertical atmospheric stability and humidity measures relevant to dangerous wildfires and pyroconvective processes. Projected changes in extreme measures of CH and FFDI are examined using a multi-method approach, including an ensemble of global climate models together with two ensembles of regional climate models. The projections show a clear trend towards more dangerous near-surface fire weather conditions for Australia based on the FFDI, as well as increased pyroconvection risk factors for some regions of southern Australia based on the CH. These results have implications for fields such as disaster risk reduction, climate adaptation, ecology, policy and planning, noting that improved knowledge on how climate change can influence extreme wildfires can help reduce future impacts of these events.

## Introduction

Strong and deep convection can sometimes occur within a fire plume, as a phenomenon known as pyroconvection, with influencing factors including weather conditions near the fire and at higher levels in the troposphere as well as the heat and moisture release by combustion^1–9^. In particular, condensation of moisture in the fire plume can release latent heat and lead to enhanced convection, with clouds formed in this way referred to as pyrocumulus (pyroCu) or pyrocumulonimbus (pyroCb) in the more intense cases where thunderstorm formation occurs. In extreme cases, this fire-atmosphere coupling can make wildfire events more dangerous, including through feedback processes between the atmosphere and the fire. These feedback processes include strong variations in surface wind direction and speed associated with convective inflows and downdrafts that can influence fire behaviour^3,10,11^ as well as pyrogenic lightning igniting new fires^5^.

Wildfires with extreme pyroconvection have recently caused severe impacts in Australia, including the deaths of many people. Examples include the Black Saturday (February 2009) and Canberra fires (January 2003) in southern Australia which had pyroconvection strong enough to produce pyroCb that injected smoke into the stratosphere^1,6,12,13^. This volcano-like process has also been documented for extreme pyroconvection events in other regions including in North America^3,7,14–16^.

The Haines Index (HI) was developed in North America to indicate the potential for vertical stability and moisture to influence fire behaviour^17^, while noting a range of other factors that can influence fire behaviour (including fire ignition sources, fuel characteristics, near-surface weather conditions and fire management activities^4,7,8,12,18^). HI was adapted for Australian conditions, including an extended upper range, given that high values occur frequently in some regions of Australia^4,8^. This extended version, known as the Continuous Haines index (CH), is used operationally by the Australian Bureau of Meteorology and fire agencies to provide guidance on lower to mid-tropospheric conditions associated with dangerous pyroconvective processes (including the formation of pyroCb events), together with the consideration of other complementary information such as fuel availability and near-surface weather conditions as well as potential ignition sources. The CH is based on a stability component (CA) and a humidity component (CB), as detailed in the Methods section.

Previous studies have examined the climatology of HI in North America^18–21^ and the Mediterranean Basin^22^, as well as of CH in Australia^4,8,23^. However, future climate projections of CH have not previously been examined for different regions throughout Australia. Future projections of near-surface fire weather conditions have been produced previously for Australia primarily based on the McArthur Forest Fire Danger Index^24^ (FFDI) as is used in this study. Here we present future projections of CH and of FFDI, based on increasing atmospheric greenhouse gas concentrations, with results mapped throughout Australia to allow regional features to be examined. An ensemble of global climate models (GCMs) is combined with two other ensembles of regional climate models (RCMs) using two different dynamical downscaling techniques. This multi-method approach is used to provide a comprehensive sample of plausible future changes, allowing near-surface fire weather conditions and risk factors associated with dangerous pyroconvection events to be examined.

## Results

### Current climate for CH

**Figure 1:**
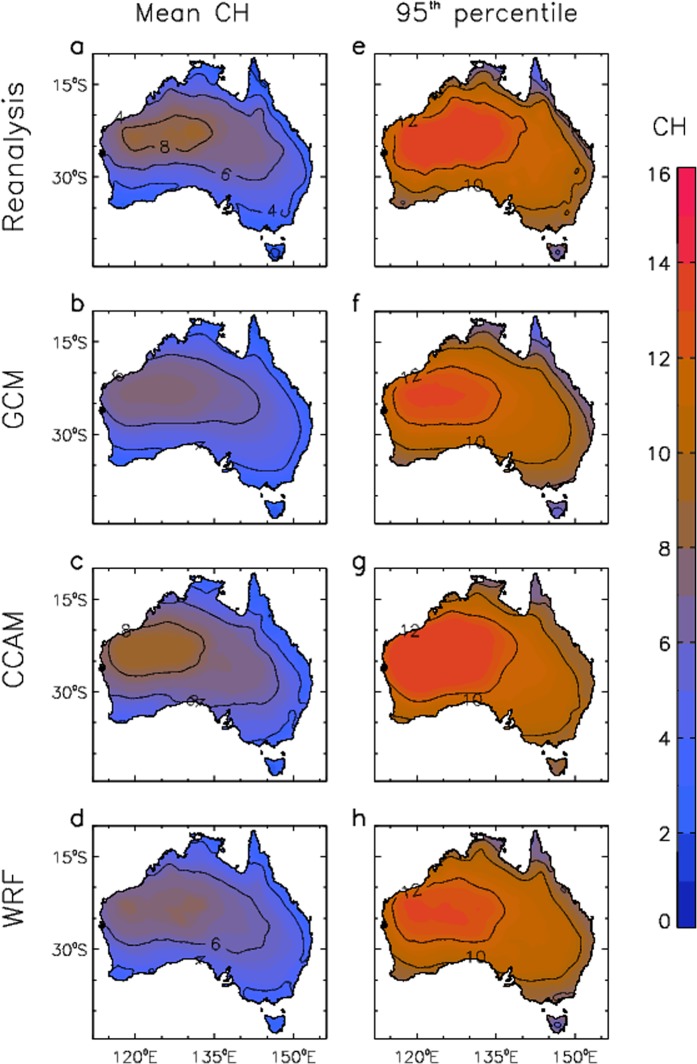
Historical pyroconvection conditions based on daily values of CH for the period 1990–2009. Mean values of CH are shown for different data sets: (**a**) ERA-Interim reanalysis; (**b**) GCMs; (**c**) CCAM; and (**d**) WRF (d). 95^th^ percentiles of CH are shown for different data sets: (**e**) ERA-Interim reanalysis; (**f**) GCMs; (**g**) CCAM; and (**h**) WRF.

Figure 1 shows the mean and 95^th^ percentile values of CH, calculated based on each day throughout the historical time period 1990–2009. Mid-afternoon values (corresponding to 0600 UT) are used throughout this study, as this is when dangerous fire conditions most often occur. Results are shown using the ERA-Interim reanalysis data^25^, which have previously been found to provide an accurate match to CH values calculated from radiosonde observations at individual stations around Australia^4,8^. Results are also shown based on three different modelling techniques including a 15-member ensemble of GCMs, an 8-member ensemble of RCM simulations using the Conformal-Cubic Atmospheric Model (CCAM) model^26,27^ and a 12-member ensemble of RCM simulations using the Weather Research and Forecasting (WRF) model^28,29^, as detailed in the Methods section. Results are calculated individually for each model, prior to calculating ensemble mean values throughout this study.

The different modelling methods are similar to the reanalysis in their representation of broad-scale features for the CH climatology (Fig. 1a–d), with higher values (above 6) in northwest and central regions and lower values (below 4) closer to the coast and in southern and eastern regions. Results for the 95^th^ percentile (Fig. 1e–h) show similar spatial variations to the results for mean values, but with higher magnitudes ranging from about 8 to 12 in most regions. Some variation between modelling approaches is apparent. In particular, the GCM and WRF results are relatively low as compared to both the ERA-Interim reanalysis and CCAM results in a northwest region of inland Australia (centred on about 125°E and 25°S), noting that this northwest region is characterised by desert regions and sparse vegetation not conducive to extreme fire behaviour associated with pyroconvection (e.g., a catalogue of over 30 pyroCb events listed for Australia has none in the northwest of Australia^5^).

**Figure 2:**
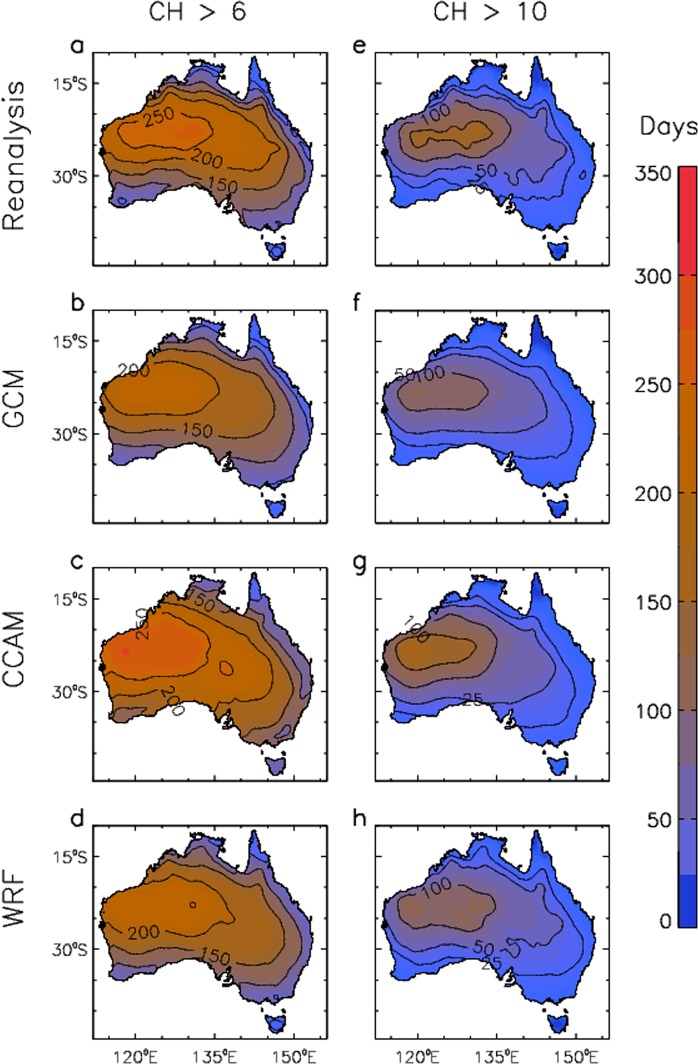
Historical extreme values of CH for the period 1990–2009. The number of days per year on average that CH exceeds a threshold value is presented. This is shown based on CH >6 for different data sets: (**a**) ERA-Interim reanalysis; (**b**) GCMs; (**c**) CCAM; and (**d**) WRF. This is also shown for CH >10 for different data sets: (**e**) ERA-Interim reanalysis; (**f**) GCMs; (**g**) CCAM; and (**h**) WRF.

Extreme measures of CH are presented in Fig. 2. For ERA-Interim reanalysis, some northwest and central regions have CH >6 more than 200 days a year, and CH >10 more than 100 days a year, with somewhat lower values in coastal regions and in southern and eastern Australia. These features are reproduced well by the GCM ensemble as well as the CCAM and WRF downscaling ensembles. There is some variation between modelling methods, including as noted previously from Fig. 1 with relatively low values in the northwest for GCM and WRF results as compared to CCAM. The degree of consistency between data sets, including in different regions throughout Australia based on the different measures presented in Figs 1, 2, helps provide confidence in the ability of the models to simulate different aspects of the CH climatology.

### Future climate for CH

**Figure 3:**
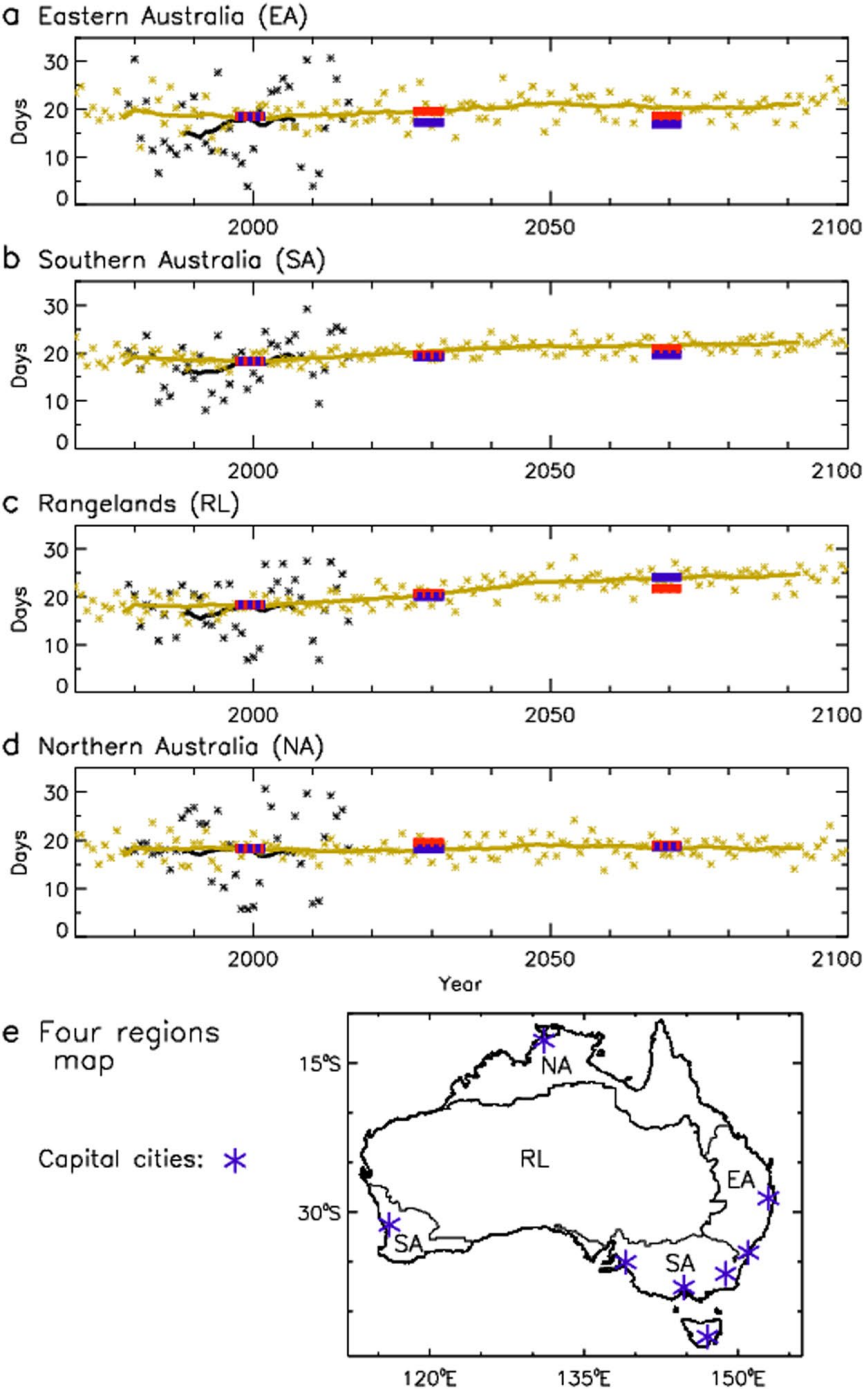
Temporal changes in pyroconvection risk for four different climatic regions. Time series showing regional trends from 1970–2100 in the average number of days with CH greater than its 95^th^ percentile at a given location. Results are presented for four regions: (**a**) Eastern Australia; (**b**) Southern Australia; (**c**) Rangelands; and (**d**) Northern Australia. (**e**) A map showing the four regions is provided, together with locations of capital cities of Australia. Results based on reanalysis cover the period 1979–2016 (shown in black) and ensemble mean values for GCMs cover the period 1970–2100 (shown in yellow), with ensemble mean values for CCAM (blue) and WRF (red) covering the periods 1990–2009, 2030–2049 and 2060–2079. A 20-year running mean (i.e., box-car average) is applied to highlight the climatological signal, with results for individual years also shown for the reanalysis and GCMs.

Figure 3 shows projected future changes in the number of days per year with CH greater than its 95^th^ percentile, with the 95^th^ percentiles defined for the historical period 1990–2009. The 95^th^ percentiles are calculated individually at each grid-cell location for each model, providing an indication of CH values that are large relative to local conditions, also noting that the 95^th^ percentiles are very high in most regions (i.e., CH is about 6 or higher from Fig. 1, with a value of 6 representing the original upper limit of the HI^17^). The use of this percentile-based threshold provides a common baseline value for the historical period, of around 18 days per year on average for all modelling methods, as a consistent reference point for examining future projected changes. The modelling methods are based on a high emissions scenario with increasing atmospheric greenhouse gas concentrations over the 21^st^ century (as detailed in the Methods section).

Area-averaged results are shown for four regions representing similar climatic conditions, as defined in previous studies^30^, including Eastern Australia (EA: a subtropical climate with maritime influences, with vegetation including large forested regions), Southern Australia (SA: a mid-latitude temperate climate, including the island of Tasmania south of the Australian continent, with vegetation including large forested regions), Rangelands (RL: a dry arid climate characterised by desert regions, with vegetation including sparsely covered grassland regions) and Northern Australia (NA: a monsoonal tropical climate, with vegetation including savanna regions with woodland and dense grassland regions). Results based on ERA-Interim reanalysis cover the period 1979–2016, with GCM results for 1970–2100. The CCAM and WRF results are shown for 1990–2009, 2030–2049 and 2060–2079 representing the common periods available for these dynamical downscaling methods. A 20-year moving average is used to highlight the climatological signal, with individual years also shown for ERA-Interim reanalysis and GCM results.

These regionally averaged projections provide some indication of a long-term climatological trend towards increased values in most cases. The largest changes later this century (for 2060–2079 as compared to 1990–2009) are for the RL region with increases ranging from 3.5–5.7 days per year for the different modelling methods, as well as for the SA region with increases ranging from 1.3–3.4 days per year. The ERA-Interim reanalysis results also indicate increasing CH values for the historical period in some cases, noting that the interannual variability is larger for the reanalysis than the GCMs given that the GCM results are based on an 18-member ensemble. There is only one example of a decrease later this century with a reduction of -1.4 days per year on average in the EA region for the CCAM results (with further details on values for individual modelling methods provided in Supplementary Table 1).

### Current climate for FFDI

Previous studies have shown that near-surface fire weather conditions as represented by the FFDI can provide complementary information to that provided by the CH in relation to the risk of dangerous wildfire and pyroconvection events in Australia^4,8^. In contrast to CH which is based on lower to mid-tropospheric stability and moisture measures, the FFDI is based on combining daily maximum temperature (at a height of 2 m) with mid-afternoon values of relative humidity (at a height of 2 m) and wind speed (at a height of 10 m) as well as a drought factor representing fuel availability (based on rainfall and temperature to indicate a temporally accumulated fuel moisture deficit). Details on the FFDI are provided in the Methods section.

**Figure 4:**
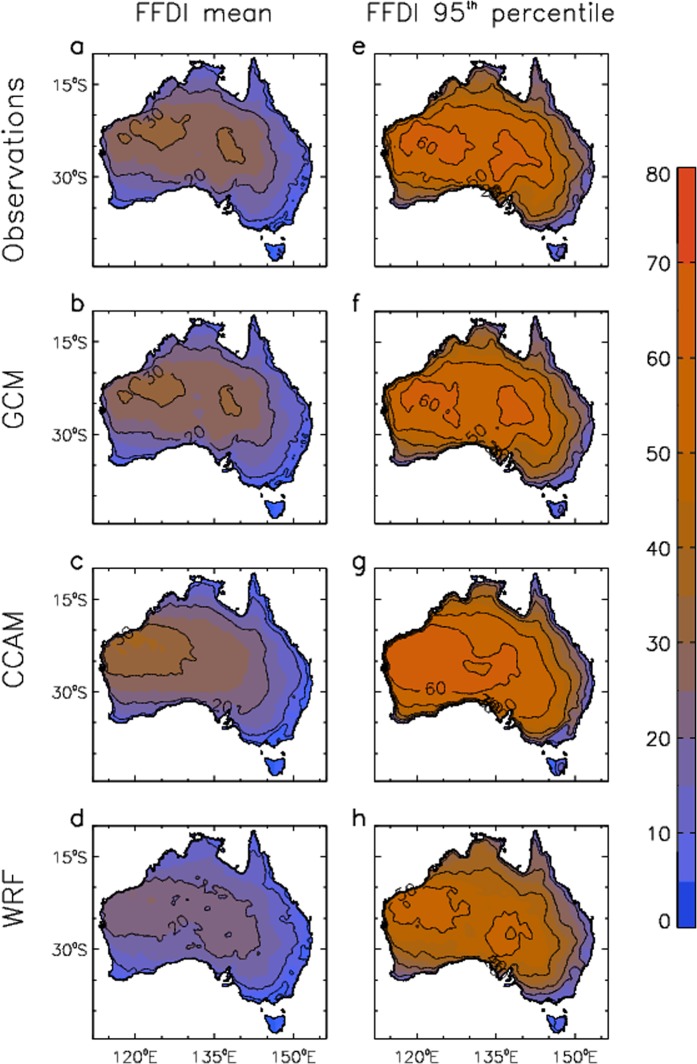
Historical near-surface fire weather conditions based on daily values of FFDI for the period 1990–2009. Mean values of FFDI are shown for different data sets: (**a**) observations-based data; (**b**) GCMs; (**c**) CCAM; and (**d**) WRF (d). 95^th^ percentiles of FFDI are shown for different data sets: (**e**) observations-based data; (**f**) GCMs; (**g**) CCAM; and (**h**) WRF.

Figure 4 shows FFDI values mapped throughout Australia for the period 1990–2009, including mean values and the 95^th^ percentile of daily FFDI. Results are shown using an observations-based data set of FFDI^31^ as well as FFDI calculated here from the same set of models as used for CH (i.e., the same ensembles for the GCM, CCAM and WRF data as described in the Methods section).

The different FFDI datasets show broadly similar results to each other, including higher values in regions further inland away from the coast in general, with the highest values occurring in the central and northwest regions of Australia. These general features of the FFDI climatology are similar to those discussed in previous studies, including based on gridded data^31^ and station observations^32^.

### Spatial detail of future projections

**Figure 5:**
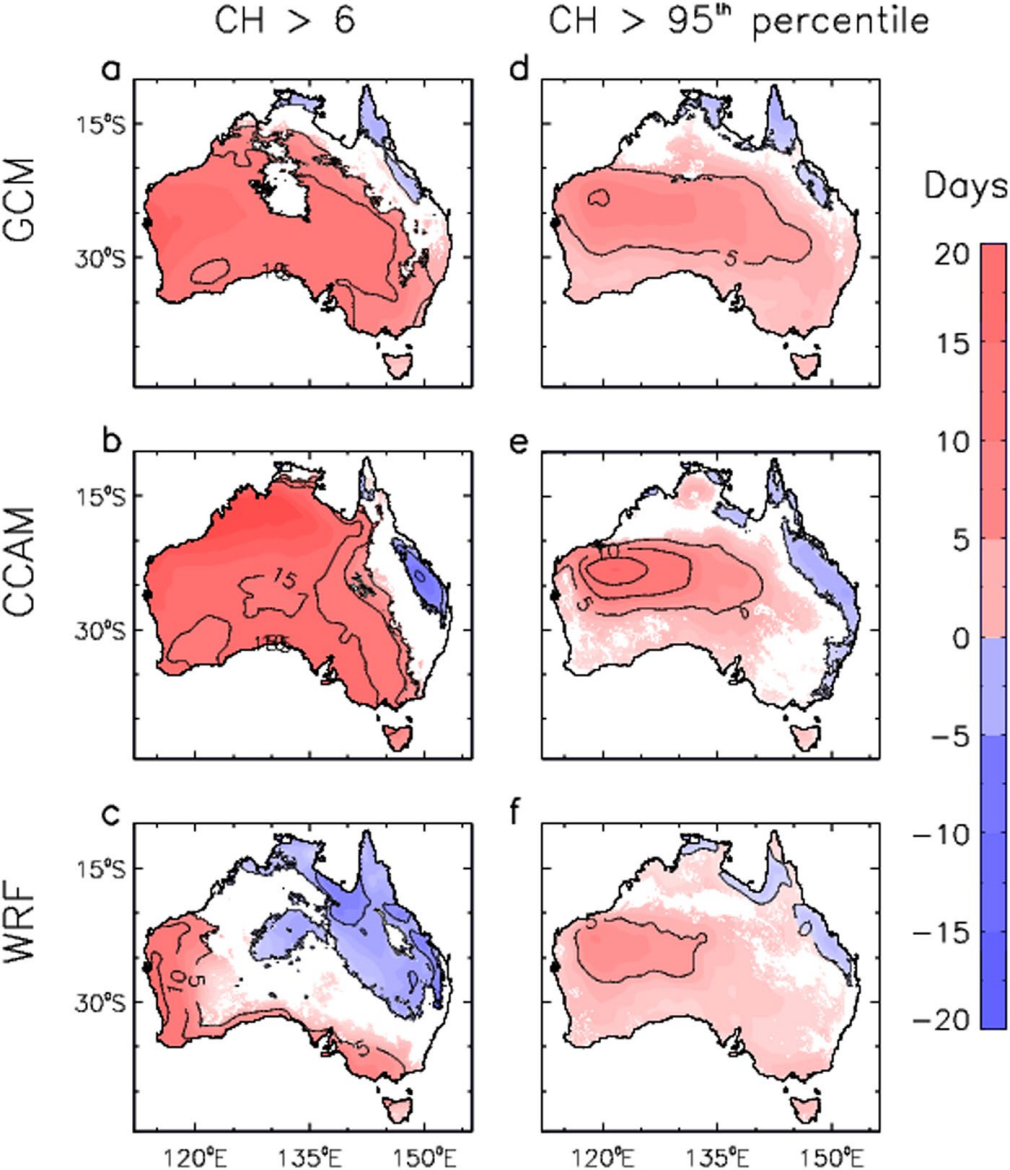
Spatial changes in pyroconvection risk for three different modelling methods. Changes are shown for the number of days per year that the CH index exceeds a threshold value, based on changes from the period 1990–2009 to the period 2060–2079. Results are presented for the number of days per year that CH is above 6 for different data sets: (**a**) GCMs; (**b**) CCAM; and (**c**) WRF. Results are also presented for the number of days per year that CH is above its historical period 95^th^ percentile for different data sets: (**d**) GCMs; (**e**) CCAM; and (**f**) WRF. Coloured regions represent locations where at least two thirds of the ensemble members for each modelling method agree of the sign of the change.

**Figure 6:**
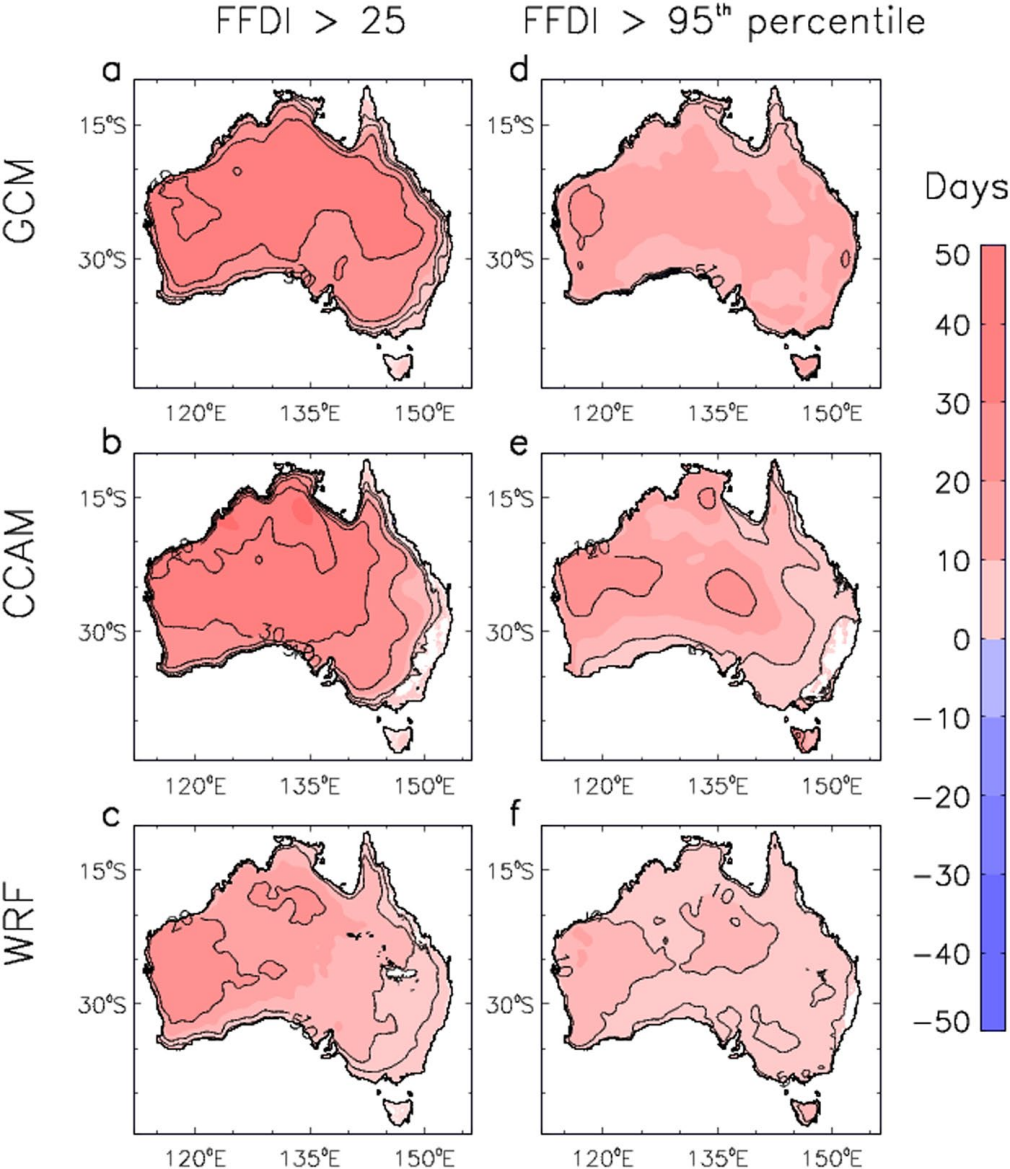
Spatial changes in near-surface fire weather condition for three different modelling methods. Changes are shown for the number of days per year that the FFDI exceeds a threshold value, based on changes from the period 1990–2009 to the period 2060–2079. Results are presented for the number of days per year that FFDI is above 25 for different data sets: (**a**) GCMs; (**b**) CCAM; and (**c**) WRF. Results are also presented for the number of days per year that FFDI is above its historical period 95^th^ percentile for different data sets: (**d**) GCMs; (**e**) CCAM; and (**f**) WRF. Coloured regions represent locations where at least two thirds of the ensemble members for each modelling method agree of the sign of the change.

Figure 5 shows spatial features throughout Australia of the projected future changes in the number of days with CH greater than 6, as well as with CH greater than its historical 95^th^ percentile (i.e., defined at each grid location for the historical period 1990–2009). Similarly, Fig. 6 shows projected future changes in the number of days with FFDI great than 25, as well as with FFDI greater than its historical 95^th^ percentile. This allows extreme values to be examined based on two complementary measures: a fixed-magnitude threshold as well as a percentile-based threshold indicating values that are high relative to local conditions. For each of the three different modelling methods, results are only shown for locations where at least two thirds of the ensemble members agree on the sign of the projected change.

There are clear spatial variations in the projected changes in CH for later this century (for the period 2060–2079 as compared to 1990–2009), with increases for many southern and western regions of Australia as well as decreases for some northeast regions. This is the case for the results based on CH >6 (Fig. 5a–c) as well as CH >95^th^ percentiles (Fig. 5d–f) for each of the three different modelling methods.

The projected increases in mid-latitude regions of southern Australia (south of about 30°S) include locations where extreme pyroconvection events have occurred, including pyroCb events near large population centres in southeast Australia (e.g., Black Saturday fires near Melbourne^6,12^) and southwest Australia (e.g., Waroona fires near Perth^11^). PyroCb events have been observed in the southern and eastern regions of Australia, such as listed in a catalogue of more than 30 occurrences identified by satellite data^5^, with no events described in the literature to date for the more northwest and central regions of Australia. For the projected increases in northwest and central Australia, it is noted that this region is sparsely vegetated in general with a low risk of dangerous pyroconvection events occurring, in contrast to southern Australia which is generally more densely vegetated and heavily populated (e.g., there are no large cities in that northwest region, with the majority of Australia population living around the capital city locations shown in Fig. 3e). For the projected decreases in the northeast, this region is characterised by wet tropical forest and is not a region where extreme pyroconvection events have been observed to date, also noting that this area of decrease is somewhat larger for the downscaling methods (CCAM and WRF) than the GCMs which may plausibly relate to their better representation of regional details (e.g., the Great Dividing Range near the east coast of Australia and the relatively narrow eastern seaboard can have distinct climate characteristics to regions further inland).

The projected increases in southern Australia are largely due to increases in the humidity component (CB) of the CH rather than the stability component (CA), with this being the case for all three modelling methods (as detailed further in Supplementary Fig. 1). This indicates a projected change towards drier conditions in the lower troposphere (i.e., increased dew point depression largely driven by increased 850 hPa temperature) as the dominant influence over changes in the temperature lapse rate (from 850–700 hPa for CA), noting that this lapse rate shows either little change or a relatively small increase in some parts of southern Australia as well as a small decrease in some other locations (such as some near-coastal southern locations and the island of Tasmania).

Figure 6 shows projected changes in the number of days with FFDI greater than 25, as well as with FFDI greater than its historical 95^th^ percentile. For each of the three different modelling methods, results are only shown for locations where at least two thirds of the ensemble members agree on the sign of the projected change. The different modelling approaches are consistent with each other in projecting a future increase in the severity of near-surface fire weather conditions as represented by the FFDI for almost all regions throughout Australia.

The projections indicate more days in the future with FFDI >25, noting that this threshold value represents conditions classed as “Very High” for operational fire management applications in Australia, as well as more days exceeding the historical 95^th^ percentile. There are some regions where less than two thirds of the models agree on the projected direction of change, particularly near the east coast for the two dynamically downscaled approaches (CCAM and WRF). Previous studies have also reported a projected future increase in near-surface fire weather conditions for different regions throughout Australia, including based on the FFDI calculated from CMIP5 GCMs^30^, while noting that this is the first time that a multi-method approach has been used (i.e., based on combining ensembles from different modelling approaches).

## Discussion

Climate change can influence conditions at different levels of the atmosphere of relevance to extreme fire weather and pyroconvection events. Recent studies have found that anthropogenic climate change has already had a significant influence on near-surface weather conditions associated with dangerous wildfires in some regions of the world including parts of North America and Australia^31,33^. In relation to lower to mid-tropospheric conditions, a long-term trend towards higher CH values in southeast Australia was reported for the historical climate based on ERA-Interim reanalysis^8^, as well as for the future climate based on WRF modelling for a single area-averaged region of southeast Australia^23^.

Our findings provide new insight into how risk factors associated with dangerous pyroconvection events (such as pyroCb activity) could change in different regions of Australia, with spatial features in the future projections of CH examined here for the first time, including based on a range of different modelling methods and different metrics of extreme conditions. Additionally, projected future changes in FFDI show broad-scale increases in the severity of near-surface fire weather throughout Australia. The region of increased severity is more widespread for FFDI than CH, highlighting the complementary information represented by these two indices.

This comprehensive approach to examining the influence of climate change on fire weather conditions in Australia helps provide enhanced confidence in the projected changes, with many regions having a consistent direction of change projected by different models and for different metrics. In particular, we find an increased risk of dangerous atmospheric conditions associated with extreme fire weather and pyroconvection for many regions of southern Australia, including in the southeast and southwest where dangerous wildfires with extreme pyroconvection have recently occurred near densely populated regions.

These findings highlight the influence that future climate change could have on the characteristics of dangerous wildfires. An improved understanding of these events is important for a range of fields, including disaster risk reduction, climate adaptation, ecology, policy and planning, in relation to reducing the damage that wildfires can cause.

## Methods

### C-Haines formulation

The C-Haines index (CH) is formulated from two components, CA and CB^4,8^, as shown by Equations 1–4. CA is the Stability Score based on the temperature difference T850 – T700, where T850 and T700 are the temperatures at 850 hPa and 700 hPa, respectively. CB is the Humidity Score based on the 850 hPa dew point depression (DD850): equal to T850 – DP850, where DP850 is the dew point temperature at 850 hPa.

1 $${\rm{CA}}=0.5\,\ast \,({\rm{T}}850-{\rm{T}}700-4)$$

2 $${\rm{CB}}=0.3333\,\ast \,({\rm{DD}}850-3),{\rm{with}}\,{\rm{DD850}}\,{\rm{limited}}\,{\rm{to}}\,{\rm{a}}\,{\rm{maximum}}\,{\rm{of}}\,{\rm{30}}\,^\circ C$$

3 $${\rm{if}}\,{\rm{CB}} > 5,{\rm{then}}\,{\rm{CB}}=5+0.5\,\ast \,({\rm{CB}}-5)$$

4 $${\rm{CH}}={\rm{CA}}+{\rm{CB}}$$

CH is calculated throughout this study using 0600 UTC values as provided by the various data sources, as this most closely aligns with mid-afternoon conditions in Australia, which is when dangerous fire activity most commonly occurs.

### FFDI formulation

The McArthur Mark V Forest Fire Danger Index^24,34^ (FFDI) is calculated as shown by Equation 5 using daily maximum temperature at a height of 2 m (*T*), mid-afternoon values of relative humidity at a height of 2 m (*RH*), mid-afternoon values of wind speed at a height of 10 m (*W*) as well as a drought factor (*DF*) representing fuel availability based on a soil moisture deficit. The Keetch Byran Drought Index^35^ (KBDI) is used here for the soil moisture deficit, calculated from daily rainfall and daily maximum temperature at a height of 2 m. For the climate model data (including the GCM, CCAM and WRF data sets), relative humidity and wind speed values at 0600 UT are used to represent mid-afternoon values for Australia. A data set of FFDI values based on a gridded analysis of observations is also used here, with a grid of 0.05 degrees in latitude on longitude throughout Australia, as detailed in a previous study^31^.

5 $$FFDI=2{e}^{(0.0338T+0.0234W\mbox{--}0.0345RH+0.987ln(DF)\mbox{--}0.45)}$$

### Reanalysis data

CH is calculated here from 1979–2016 based on ERA-Interim reanalysis^25^ with a temporal resolution of 6 hours and a grid spacing of 0.75 degrees in both latitude and longitude throughout Australia. This 0.75-degree grid is also used for the analysis of the model results presented throughout this study, with each individual model bilinearly interpolated to the grid used by the reanalysis to provide a consistent spatiotemporal framework.

### GCM data

In conjunction with the Intergovernmental Panel on Climate Change (IPCC), a set of GCM experiments has been produced: the World Climate Research Program Coupled Model Intercomparison Project phase 5 (CMIP5)^36^. The direct output from 15 CMIP5 GCMs is used here to examine CH values, selected based on availability of 6-hourly data archived on Australia’s national repository of CMIP5 data. The GCMs are ACCESS1-0, ACCESS1-3, BCC-CSM1–1, BCC-CSM1–1-M, BNU-ESM, CCSM4, CNRM-CM5, CSIRO-Mk3-6-0, FGOALS-G2, GFDL-CM3, GFDL-ESM2G, GFDL-ESM2M, MIROC5, MRI-CGCM3 and NorESM1-M. These 15 GCMs were also used to examine FFDI values, with the four weather variables used for the FFDI calculation (temperature, relative humidity, wind speed and rainfall) first being calibrated using quantile matching to be more consistent with the input variables used for the observations-based FFDI data set^31,37^. A high emission pathway is considered here (RCP8.5, with no stabilization this century leading to about 1370 ppm CO_2_ equivalent by 2100^38^) to examine the influence of increased greenhouse gas concentrations.

### CCAM downscaling

The CCAM method is based on correcting the bias and variance of GCM Sea Surface Temperatures (SSTs), with details and model performance as described in recent studies^26,27,30,39^. A 50 km resolution global CCAM simulation is performed using the corrected GCM SSTs as a boundary condition. Changes in the SSTs due to global warming as simulated by the GCMs are then preserved by this technique, although the simulation biases during the present climate are reduced due to using the corrected GCM SSTs. CCAM used this corrected SSTs technique to downscale eight GCMs from CMIP5 for the high emissions pathways (RCP8.5). The GCMs selected were ACCESS1-0, CNRM-CM5, GFDL-ESM2M, HADGEM2, MIROC5, NorESM1-M, CanESM2 and CESM1-CAM5.

### WRF downscaling

The WRF downscaling data used here were produced by the NSW and ACT Regional Climate Modelling (NARCliM) project which was designed to create regional-scale climate projections over south-eastern Australia for use in climate change impacts and adaptation studies^29^. Three RCMs were used to downscale four GCMs for three 20-year time slices: 1990–2009, 2020–2039 and 2060–2079. For future projections, the Special Report on Emissions Scenario (SRES) A2 emission scenario was used, representing a high emission pathway based on the Coupled Model Intercomparison Project phase 3 (CMIP3) set of GCM experiments. The GCMs chosen were MIROC3.2, ECHAM5, CCCMA3.1 and CSIRO-MK3.0 and the RCMs were different configurations of the WRF model^28^ with different parameterizations of planetary boundary layer, surface layer, cumulus physics, microphysics and radiation^29^. In general, it has been found to perform well, apart from a small wet and cold bias overall. In a comprehensive analysis it was found that the NARCliM ensemble provided measurable added-value over the driving GCMs^40^.

## Supplementary information

Supplementary information
