# Supplementary material for: Future changes in extreme weather and pyroconvection risk factors for Australian wildfires
Source: Sci Rep. 2019 Jul 11;9:10073. doi: 10.1038/s41598-019-46362-x (PMC6624367; doi:10.1038/s41598-019-46362-x)
Supplement: Supplementary file 1 — Supplementary information [file 41598_2019_46362_MOESM1_ESM.docx]

**Supplementary Information:**

**Future changes in extreme weather and pyroconvection risk factors for Australian wildfires**

**Andrew J. Dowdy^1*^, Hua Ye^1^, Acacia Pepler^1^, Marcus Thatcher^2^, Stacey L. Osbrough^2^, Jason P. Evans^3,4^, Giovanni Di Virgilio^3^, Nicholas McCarthy^5^**

^1^Climate Research Section, Bureau of Meteorology, Melbourne, Australia

^2^CSIRO, Melbourne, Australia

^3^Climate Change Research Centre, University of New South Wales, Sydney, Australia

^4^Australian Research Council Centre of Excellence for Climate Extremes, University of New South Wales, Sydney, Australia

^5^University of Queensland, Brisbane, Australia

*Correspondence to: [andrew.dowdy@bom.gov.au](mailto:andrew.dowdy@bom.gov.au); Bureau of Meteorology, 700 Collins St, Docklands, VIC, Australia.

**Additional detail on NARCliM modelling method**

The NARCliM project was designed to create regional-scale climate projections for use in climate change impacts and adaptation studies, and ultimately to inform climate change policymaking^29^. Details can be found on the AdaptNSW website (<http://climatechange.environment.nsw.gov.au/Climate-projections-for-NSW/About-NARCliM>).

In NARCliM three RCMs are used to downscale four GCMs for three 20-year time slices (1990–2009 or ‘present’; 2020–2039 or ‘near future’; 2060–2079 or ‘far future’). For future projections, the Special Report on Emissions Scenario (SRES) A2 emission scenario is used. A careful choice of both RCMs and GCMs was made to ensure all models performed adequately^41,42^, were independent^29^ and the GCMs spanned the future change range of temperature and precipitation. The GCMs chosen are the MIROC3.2, ECHAM5, CCCMA3.1 and CSIRO-MK3.0. The chosen RCMs are versions of the WRF model with different parameterizations of planetary boundary layer, surface layer, cumulus physics, microphysics and radiation. The NARCliM ensemble has been evaluated for its ability to simulate the mean climate^43^, precipitation and temperature extremes^44,45^, large-scale climate mode teleconnections^46^, and dynamical features such as low pressure systems^47^. In general it has been found to perform well with a small wet and cold bias overall. In a comprehensive analysis found that the NARCliM ensemble provided measurable added-value over the driving GCMs^40^.

**Details of projected regional changes for individual modelling approaches**

Specific values are presented in Supplementary Table 1, based on the results presented in Fig. 3, with values listed for each individual modelling approach for the four regions.

**Supplementary Table 1. Projected changes in the annual mean number of days with CH exceeding its 95^th^ percentile at a given location.** Values are listed here for each individual modelling approach (i.e., the GCMs, CCAM and WRF ensembles), presented individually for each of the four regions examined in this study (Eastern Australia ‘EA’, Southern Australia ‘SA’, Rangelands ‘RL’ and Northern Australia ‘NA’). These results are based on data as shown in Figures 3 and 4.

| **Region** | **GCMs** | **CCAM** | **WRF** |
| --- | --- | --- | --- |
| EA | 2.4 | -1.4 | 0.3 |
| SA | 3.4 | 1.3 | 2.5 |
| RL | 5.7 | 5.7 | 3.5 |
| NA | 0.2 | 0.3 | 0.7 |

**Additional detail on input variables to CH**

To provide further insight into the projected changes for the different modelling methods, Supplementary Fig. 1 shows how the Stability Score (CA) and the Humidity Score (CB) are projected to change. CA is based on the mid-tropospheric temperature lapse rate (from 850-700 hPa), calculated following Equation 1 in the Methods section. CB is based on the 850 hPa dewpoint depression, calculated following Equations 2-3 in the Methods section.

**
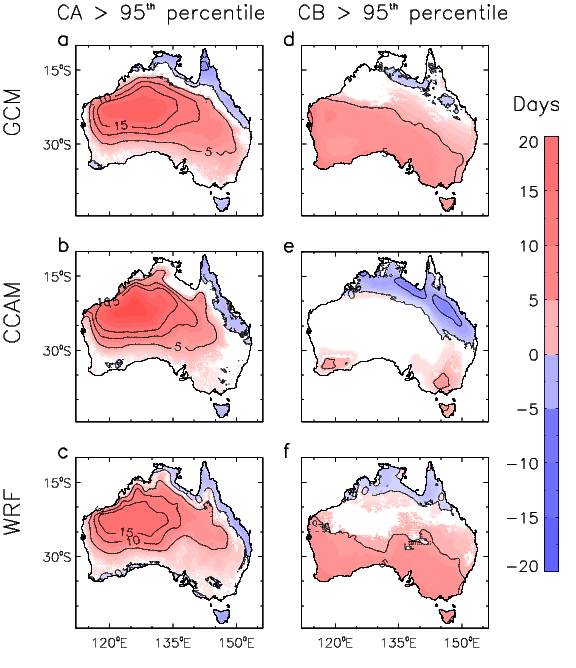
**

**Supplementary Fig. 1. Spatial changes in CA and CB for three different modelling methods.** Percentage changes are shown for the number of days per year that the indices exceed a threshold value, based on changes from the period 1990–2009 to the period 2060–2079. Results are presented for the number of days per year that CA is above its historical period 95^th^ percentile for different data sets: **a**, GCMs; **b**, CCAM; and **c**, WRF. Results are also presented for the number of days per year that CB is above its historical period 95^th^ percentile for different data sets: **d**, 95^th^ percentile for GCMs; **e**, CCAM; and **f**, WRF. Coloured regions represent locations where at least two thirds of the ensemble members for each modelling method agree of the sign of the change.

**References (continued numbering from primary manuscript):**

1. Evans, J., Ekström, M. & Ji, F. Evaluating the performance of a WRF physics ensemble over South-East Australia. *Climate Dynamics* **39**(6), 1241–1258, <http://dx.doi.org/10.1007/s00382-011-1244-5> (2012).
2. Ji, F., Ekström, M., Evans, J. P. & Teng, J. Evaluating rainfall patterns using physics scheme ensembles from a regional atmospheric model. *Theoretical and Applied Climatology* **115**(1–2), 297–304 (2014).
3. Olson, R., Evans, J. P., Luca, A. D., Argeso, D. The NARCliM project: model agreement and significance of climate projections. *Climate Research* **69**(3), 209–227, <http://dx.doi.org/10.3354/cr01403> (2016).
4. Evans, J. P., Argueso, D., Olson, R. & Luca, A. D. Bias-corrected regional climate projections of extreme rainfall in south-east Australia. *Theoretical and Applied Climatology* **130**(3–4), 1085–1098 (2017).
5. Evans, J. P., Argueso D. & Di Luca, A. D. *Future heatwaves in NSW from the NARCliM ensemble*. In Syme, G., Hatton MacDonald, D., Fulton, B. and Piantadosi, J. (eds) MODSIM2017, 22nd International Congress on Modelling and Simulation. Modelling and Simulation Society of Australia and New Zealand, December 2017, 1208–1214 (2017).
6. Fita, L., Evans, J. P., Argüeso, D., King, A. & Liu, Y. Evaluation of the regional climate response in Australia to large-scale climate modes in the historical NARCliM simulations. *Climate Dynamics* **49**, 2815-2829 (2017).
7. Di Luca, A., Evans, J. P., Pepler, A., Alexander, L. V. & Argueso, D. Australian East Coast Lows in a Regional Climate Model ensemble. *Journal of Southern Hemisphere Earth Systems Science* **66**, 108–124 (2016a).
